# Organic and Functional Nervous Diseases

**Published:** 1910-01

**Authors:** 


**Organic and Functional Nervous Diseases.** A Textbook of Neurology. By **M. Allen Starr,** M.D., Ph.D., LL.D., Sc.D., Professor of Neurology, College of Physicians and Surgeons, New York. Third edition. Octavo, 904 pages, with 300 engravings and 29 plates in colors or monochrome. Lea & Febiger, Philadelphia and New York, 1909. (Cloth, $6.00; leather, $7.00, net prices.)

The third edition of this book is presented in four parts,—a very convenient arrangement for reference. Part one has to do with the general aspect of neurology, methods of examination and diagnosis, with the necessary anatomy and physiology. Part two takes up the organic diseases, with such additions as has been made to our knowledge of the subject during the past three years.

In part three functional diseases are fully presented, the space allotted for a discussion of these subjects having been more than doubled. A new chapter on reflex neurosis has been added. Part four takes up diseases of the sympathetic nervous system.

The author has taken advantage of this opportunity to make some changes in the arrangement of the contents of the book, and it is evident that he has given it a careful revision. He presents his views in a terse yet pleasant and readable manner. The book covers all aspects of neurology, both medical and surgical. The work is a credit to its author. J. A. R.

